# Different glomerular filtration rate estimating formula for prescribing DOACs in oldest patients: appropriate dosage and bleeding risk. Post hoc analysis of a prospective cohort

**DOI:** 10.1007/s40520-021-01986-w

**Published:** 2021-10-18

**Authors:** Valeria Calsolaro, Chukwuma Okoye, Sara Rogani, Alessia Maria Calabrese, Umberto Dell’Agnello, Rachele Antognoli, Daniela Guarino, Fabio Monzani

**Affiliations:** Geriatrics Unit, Department of Clinical and Experimental Medicine, University Hospital of Pisa, Via Paradisa 2, 56124 Pisa, Italy

**Keywords:** Glomerular filtration rate, Chronic kidney disease, Direct oral anticoagulants, Atrial fibrillation, Serum creatinine, Cystatin-C

## Abstract

**Background:**

Direct oral anticoagulants (DOACs) pharmacokinetics depends on estimated glomerular filtration rate (eGFR), whose estimation is crucial for optimal risk/benefit balance.

**Aims:**

To assess the concordance among different eGFR formulas and the potential impact on DOACs prescription appropriateness and bleeding risk in oldest hospitalized patients.

**Methods:**

Post hoc analysis of a single-centre prospective cohort study. eGFR was calculated by creatinine-based (MDRD, CKD-EPI_Cr_, BIS_1_) and creatinine–cystatin-C-based (CKD-EPI_Comb_ and BIS_2_) formulas. Patients were stratified according to eGFR [severely depressed (SD) 15–29; moderately depressed (MD) 30–49; preserved/mildly depressed (PMD): ≥ 50 ml/min/1.73 m^2^]. Concordance between the different equations was assessed by Cohen’s kappa coefficient.

**Results:**

Among AF patients, 841 (59.2% women, mean age 85.9 ± 6.5 years) received DOACs. By CKD-EPI_Cr_ equation, 135 patients were allocated in the SD, 255 in the MD and 451 in the PMD group. The concordance was excellent only between BIS 2 and CKD-EPI_Comb_ and MDRD and CKD-EPI_Cr_, while was worse (from good to poor) between the other formulas. Indeed, by adding cystatin-C almost over 1/3 of the patients were reallocated to a worse eGFR class. Bleeding prevalence increased by 2–3% in patients with discordant eGFR between formulas, reallocated to a worse chronic kidney disease (CKD) stage, although without reaching statistical significance. CKD-EPI_Comb_ resulted the best predictor of bleeding events (AUROC 0.71, *p* = 0.03).

**Discussion:**

This study highlights the variability in CKD staging according to different eGFR formulas, potentially determining inappropriate DOACs dosing. Although the cystatin-C derived CKDEPI_Comb_ equation is the most accurate for stratifying patients, BIS_1_ may represent a reliable alternative.

**Supplementary Information:**

The online version contains supplementary material available at 10.1007/s40520-021-01986-w.

## Introduction

Multimorbidity is a common problem in elderly population, especially above 85 years of age, leading to increased drug prescription and amount of adverse events, drug–drug and drug–disease interactions and inappropriate prescribing [1]. Chronic kidney disease (CKD) shows huge increase with ageing [2, 3]; the global prevalence of 1–5 CKD stages has been estimated to be 16–33% in subjects aged 70–74 years up to 66–93% in those older than 90 years [4]. CKD increases the risk of atrial fibrillation (AF), being its incidence from 6% in CKD 1 stage up to 36% in stage 4 [5]. Moreover, patients with CKD have increased risk of thrombotic and haemorrhagic events, especially for estimated glomerular filtration rate (eGFR) < 30 ml/min/1.73 mq [6]. Given that premise, it appears clear that the optimal management of thromboprophylaxis in patients with CKD and AF is complex, and several clinical aspects should be considered, especially multimorbidity and polytherapy, to guarantee the better risk/benefit balance. Since all direct-acting oral anticoagulants (DOACs) depend on renal excretion for elimination (with a variable amount that ranges from 27% for apixaban to 80% for dabigatran) [7], an accurate glomerular filtration rate estimation is crucial, given that 43% of patients with AF and CKD resulted potentially overdosed with DOACs [7, 8]. Kidney Disease: Improving Global Outcomes (KDIGO) recommends the creatinine-based Chronic Kidney Disease Epidemiology (CKD-EPI) equation, but eGFR may be overestimated in older patients hospitalized for acute disease [9]. Current guidelines [10] recommend the Cockroft–Gault (CG) formula for DOACs prescription although its accuracy declines at extreme age ranges [11]. Cystatin-C-based equations are thought to be more accurate in patients with low body mass index [12] and CKD-EPI equation combining creatinine and cystatin C (CKD-EPI_Comb_) is regarded as the most accurate estimator of kidney function in community-dwelling older population [13–15]. In the last years new eGFR equations, such as Modification of Diet in Renal Disease (MDRD) and Berlin Initiative Study (BIS) have been validated, but only the latter in people older than 70 years [3, 16–18]. So far, no studies have evaluated the impact of the diverse eGFR equations on DOACs management in very old patients, hospitalized for acute disease. In the current study, we aimed at assessing the level of concordance among different eGFR estimating equations and the possible impact of discordant eGFR on DOACs prescription appropriateness and clinically relevant bleedings in older, hospitalized AF patients receiving DOACs.

## Patients and methods

The study population comes from a large dataset of oldest patients with AF, hospitalized for acute medical illness [19]. Here we report on the subgroup of patients receiving DOAC therapy at hospital discharge. The study protocol complied with the Declaration of Helsinki and was approved by the Pisa University Hospital Ethic Committee. Each patient gave written informed consent to participate to the study; the legally authorized delegate provided informed consent in case of patients who were temporarily or permanently without capacity. Demographic characteristics and clinical history along with physical and functional examination, including Comprehensive Geriatric Assessment (CGA), were obtained on the day of hospital admission as previously described [19]. CGA was performed using the following scales: Cumulative Illness Rating Scale (CIRS-c) [20], Basic (ADL) and Instrumental (IADL) Activities of Daily Living [21], Short Portable Mental Status Questionnaire (SPMSQ) [22] and Mini Nutritional Assessment (MNA) [categorized in dichotomous variables (malnutrition or risk of malnutrition and normal)] [23]. The HAS-BLED [24] and CHADS2-VASc [25] scores were also calculated.

Laboratory tests were performed at admittance after an overnight fast. Circulating creatinine and cystatin-C were measured by Roche Analyzer, at the central laboratory of the University Hospital of Pisa; eGFR (ml/min/1.73 mq) was calculated according to MDRD, CKD-EPI_Cr_, CKD-EPI_comb_, and BIS_1_ and BIS_2_ formulas, using either creatinine alone or creatinine and cystatin-C values. We decided not to use Cockcroft–Gault formula due to the described eGFR underestimation in frail, older individuals, especially those hospitalized for acute heart failure (HF) [26]. According to KDIGO recommendation, CKD-EPI_Cr_ was used as the reference eGFR estimating formula and patients stratified in three categories of kidney function as follows: severely depressed (SD; eGFR 15–29 ml/min/1.73 m^2^); moderately depressed (MD; eGFR 30–49 ml/min/1.73 m^2^); preserved/mildly depressed (PMD; eGFR ≥ 50 ml/min/1.73 m^2^). The cut-offs were chosen according to the current guidelines [10], and patients with eGFR below 15 ml/min/1.73 m^2^, in whom DOACs are contraindicated, were excluded from the analysis. At hospital discharge, the prescription or not of DOACs therapy and the concordance or discordance in the assignment of patients to each eGFR class in relation to the diverse estimating formulas as compared to CKD-EPI_Cr_ was recorded. During follow-up, the treatment was verified in case of readmission to the emergency department (ED) for clinically relevant bleeding. Each ED readmission was found through a dedicated software (First Aid^®^), where reasons for access, current therapy and clinical work up are recorded, providing a reliable and detailed set of information.

### Statistical analysis

Statistical analysis was performed using SPSS 21.0 statistical software package (SPSS Inc., Chicago, IL). Continuous variables are expressed as mean ± standard deviation, ordinal variables as median and range, and categorical variables as percentage. Spearman correlation was used for either continuous or dichotomous variables. Analysis of variance (ANOVA) was used to compare continuous normally distributed variables, whereas *X*^2^ test was used for categorical or dichotomous variables. The Kruskal–Wallis test was used for not normally distributed parameters. Concordance between the various proposed equations, considered as the degree of agreement between the two formulae was evaluated by Cohen’s kappa which evaluates the consistency, considering the concordance due to the case. The coefficient of concordance (*K*) evaluates the performance of a formula compared to another (which can represent the clinical gold standard). Concordance is defined as follows: *K* 0.00–0.20 poor, 0.21–0.40 modest, 0.41–0.60 moderate, 0.61–0.80 good, 0.81–1.00 excellent [27]. The discriminatory performance of the different eGFR formulas was evaluated considering the receiver operating characteristic (ROC) curve [28]. Moreover, to evaluate the possible non-linear relationships with bleedings, a log-binomial regression, applying restricted cubic spline function, was used. Tests were performed considering a level of significance of 5%.

## Results

Demographic and clinical characteristics of the whole cohort of patients (*n* = 5093) were previously described [19]. Among patients with AF, 926 received DOACs therapy at hospital discharge; cystatin-C was not measured in 85 of them, thus all the eGFR formulas were available in 841 patients (mean age 85.9 ± 6.5 years, 59.2% women): 287 (34.1%) received rivaroxaban, 278 (33.0%) apixaban, 252 (30.0%) dabigatran and 24 (2.9%) edoxaban. Table 1 summarizes the baseline socio-demographic characteristics of the study cohort.

**Table 1:** Baseline socio-demographic and clinical characteristics of the whole study cohort (*n* = 841)

Age (years)	85.9 ± 6.5
Women (%)	496 (59.0)
Length of stay (days)	5.8 ± 2.9
CIRS-C (median, IQR)	6 (2)
ADL (median, IQR)	4 (5)
IADL (median, IQR)	2 (5)
SPMSQ (median, IQR)	2 (4)
MNA (no. of malnourished or at risk, %)	269 (32.0)
CKD-EPI_creat_ (mL/min/1.73 m^2^)	51.5 ± 23.8
History of falls (%)	123 (14.6)
Prior stroke/TIA (%)	196 (23.3)
Positive bleeding history (%)	175 (20.8)
Systemic hypertension (%)	538 (64.2)
CAD (%)	134 (15.9)
Heart failure (%)	345 (41.0)
Diabetes mellitus (%)	171 (20.2)
CHA_2_DS_2_VASc (median, IQR)	5 (4–6)
HAS-BLED (median, IQR)	2 (1–2)

Data are expressed as mean ± SD for age, length of stay, CIRS-C and CKD-EPI and as median (interquartile range—IQR) for ADL, IADL, SPMQS, CHA2DS2VASc and HAS-BLED score

*CIRS-C* Cumulative Illness Rating Scale-Comorbidity, *ADL* activities of daily living, *IADL* instrumental activities of daily living, *SPMSQ* Short Portable Mental Status Questionnaire, *MNA* mini-nutritional assessment, *CKD-EPI* chronic kidney disease-epidemiology collaboration, *CAD* coronary heart disease, *CHA*_*2*_*DS*_*2*_*VASc score* Congestive Heart Failure, Hypertension, Age ≥ 75 (Doubled), Diabetes Mellitus, Prior Stroke or Transient Ischemic Attack (Doubled), Vascular Disease, Age 65–74, Female, *HAS-BLED score* Hypertension, Abnormal renal/liver function, Stroke, Bleeding History or predisposition, Labile INR, Elderly, Drugs/alcohol concomitantly

Patients’ reallocation in each eGFR group—according to the different equations—is described in Table 2. According to the CKD-EPI_Cr_ formula, 135 (16.1%) patients were allocated in the SD group, 255 (30.3%) in the MD group and 451 (53.6%) in the PMD group. While comparing CKD-EPI_Cr_ with the other creatinine-based equations, we observed 6.7% and 35.3% (with MDRD and BIS_1_, respectively) global mismatch in the prevalence of patients across the eGFR classes. Using CKD-EPI_Comb_ as the reference equation, we observed 42.8%, 31.7% and 19.8% overall mismatch (with MDRD, CKD-EPI_Cr_ and BIS_1_, respectively). By introducing cystatin-C in the CKD-EPI formula we obtained a cumulative 15.8% and 17.4% increase in the percentage of patients with eGFR below 50 ml/min/1.73 m^2^ as compared to CKD-EPI_Cr_ and MDRD, respectively, while, by comparing BIS_1_ and BIS_2_ formulas, the mismatch was almost halved (7.7%). In detail, with CKD-EPI_Comb_ the eGFR of 16 patients (11.6%) in the SD group resulted below 15 ml/min/1.73 m^2^, while with BIS_2_ the shift was less remarkable (2.3%). Almost 1/3 of the patients shifted from the MD to the SD group by utilizing cystatin-C formulas (CKD-EPI_Comb_ and BIS_2_, 33.5% and 28.8%, respectively). Moreover, with BIS_2_ formula almost half of the patients shifted from the PMD to the MD group, whilst almost 1/3 with CKD-EPI_Comb_ and BIS_1_ equations. Thus, several patients were taking inappropriate DOAC dosage (Supplementary Table 1A) or should discontinue DOACs (Supplementary Table 1B).

**Table 2:** Patients’ reallocation in each glomerular filtration rate class according to different estimating formulas

CKD-EPI_Cr_	CKD-EPI_Comb_	BIS_1_	BIS_2_	MDRD
eGFR 15–29 (n = 135)	***16 (11.6%)*** *114 (84.5%)* **5 (3.9%)**	***0*** *125 (92.2%)* **10 (7.8%)**	***3 (2.3%)*** *125 (92.5%)* **7 (4.9%)**	***0*** *103 (76%)* **32 (24%)**
eGFR 30–49 (n = 255)	***85 (33.5%)*** *162 (63.6%)* **8 (3%)**	***2 (0.8%)*** *253 (99.2%)* **0**	***73 (28.8%)*** *179 (69.9%)* **3 (1.3%)**	***0*** *230 (90.3%)* **25 (9.7%)**
eGFR > 50 (n = 451)	0 ***150 (33.2%)*** *301 (66.8%)*	0 ***137 (30.4%)*** *314 (69.6%)*	0 ***210 (46.6%)*** *241 (53.4%)*	0 ***1 (0.3%)*** *450 (99.7%)*

Italics: remained in the same eGFR class; Bold: moved to the one-degree better eGFR class; Bold italics: moved to one-degree worse eGFR class

*eGFR* estimated glomerular filtration rate, *CKD-EPI*_*Cr*_ Chronic Kidney Disease-Epidemiology Collaboration creatinine-based, *CKD-EPI*_*Comb*_ Chronic Kidney Disease-Epidemiology Collaboration creatinine–cystatin-C based, *BIS*_*1*_ Berlin Initiative Study creatinine, *BIS*_*2*_ Berlin Initiative Study creatinine–cystatin-C, *MDRD* modification of diet in renal disease

The concordance of the three classes of kidney function in relation to the diverse eGFR equations, as assessed by Cohen’s Kappa coefficient, is shown in Supplementary Table 2. Concordance was excellent only between BIS _2_ and CKD-EPI_Comb_ (K coefficient 0.868 (IC 95% 0.844–0.891)) and between MDRD and CKD-EPI_Cr_ (K coefficient 0.915 (IC 95% 0.895–0.935)), while was poor between MDRD and BIS_2_ [weighted *K* coefficient 0.575 (IC 95% 0.536–0.614)], and either good or moderate between the other formulas.

### Bleeding events

During follow-up [median 33.4 months (95% CI 32.2–34.6)], 103/841 (12.3%) AF patients receiving DOACs were readmitted to the ED for clinically relevant bleeding; gastrointestinal system (52 patients, 50.5%) resulted the most frequent site, followed by genitourinary tract (40 patients, 38.8%), while intracranial bleedings were observed in 11 patients (10.7%). Hospitalization was necessary in all the intracranial bleedings and in half of the others, with only one bleeding-derived death during hospitalization (patient with massive intracranial bleeding, and concordant eGFR).

**Fig. 1:**
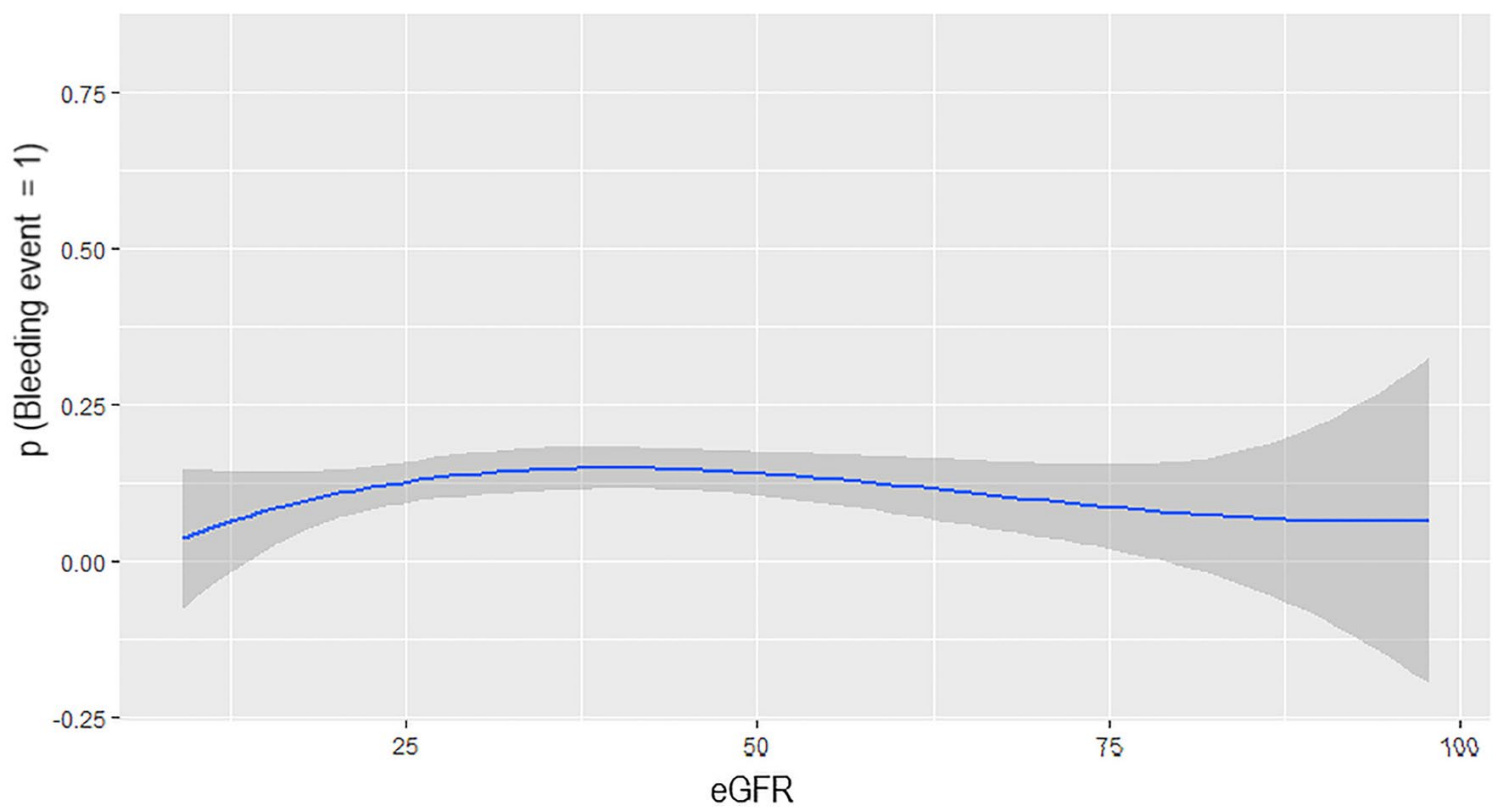
Log-binomial regression between eGFR values and bleeding events

The risk of bleeding did not significantly differ among patients in the three eGFR groups regardless the estimating formula used (Table 3). However, the incidence of bleeding events increased for eGFR values ranging from 25 ml/min/1.73 m^2^ up to 36 ml/min/1.73 m^2^ (*p* = 0.046), with a trend towards statistical significance in the third knots (eGFR ranging from 37 and 50 ml/min/1.73 m^2^, *p* = 0.053) also confirmed after adjustment for age, sex, burden of comorbidities (CIRS) and inappropriate dosage/prescription. Accordingly, a non-linear relationship was observed between eGFR value and bleeding events (Fig. 1). Moreover, using CKD-EPI_Cr_ as the reference equation, the overall prevalence of clinically relevant bleeding resulted higher, although not reaching the statistical significance, in patients with discordant eGFR, who moved to a worse group of renal function by BIS_1_, CKD-EPI_Comb_ and BIS_2_ formulas as compared to those with concordant eGFR (13.6% vs 11.7% p = 0.30, 14.3% vs 11.2% p = 0.22 and 13.8% vs 11.5% p = 0.29, respectively). Also, by comparing eGFR estimating formulas with and without cystatin-C value in the algorithm, CKD-EPI_Comb_ showed the highest accuracy in predicting bleeding events [AUROC 0.71 (CI 95% 0.66–0.74) vs 0.60 (CI 95% 0.56–0.65), 0.58 (CI 95% 0.53–0.65) and 0.51 (CI 95% 0.46–0.55) with BIS_2_, BIS_1_ and CKD-EPI_Cr_, respectively, *p* = 0.03] and, no statistical difference was found between BIS_1_ and BIS_2_ AUROC (*p* = 0.67). Accordingly, the patients (147/841, 17.5%) receiving inappropriate DOAC dosage/prescription while using CKD-EPI_Comb_ derived eGFR, showed a higher prevalence of bleeding events compared to the counterparts, although without reaching the statistical significance (13.8% vs 11.5%, *p* = 0.288).

**Table 3:** Bleeding risk across eGFR classes according to the diverse eGFR estimating formulas

	eGFR < 30		eGFR 30–50		eGFR > 50		*p* value
	Bleeding	Not bleeding	Bleeding	Not bleeding	Bleeding	Not bleeding	
CKD-EPI_Comb_	23 (11.6)	176 (88.4)	42 (13.5)	270 (86.5)	38 (12.3)	271 (87.7)	0.57
CKD-EPI_Cr_	13 (9.6)	122 (90.4)	29 (11.3)	226 (88.7)	61 (13.5)	390 (86.5)	0.17
MDRD	10 (9.7)	93 (90.3)	28 (10.7)	233 (89.3)	65 (13.7)	410 (86.3)	0.21
BIS_1_	11 (8.7)	116 (91.3)	49 (12.6)	341 (87.4)	43 (13.7)	271 (86.3)	0.19
BIS_2_	19 (9.6)	179 (90.4)	54 (13.6)	342 (86.4)	30 (12.3)	214 (87.7)	0.41

*eGFR* estimated glomerular filtration rate, *CKD-EPI*_*Cr*_ Chronic Kidney Disease-Epidemiology Collaboration creatinine-based, *CKD-EPI*_*Comb*_ Chronic Kidney Disease-Epidemiology Collaboration creatinine–cystatin-C based, *BIS*_*1*_ Berlin Initiative Study creatinine, *BIS*_*2*_ Berlin Initiative Study creatinine–cystatin-C, *MDRD* modification of diet in renal disease

## Discussion

In this post hoc analysis of a single-centre prospective cohort study [19], we have evaluated the concordance/discordance between different formulas to estimate glomerular filtration rate. The impact of discordant eGFR on DOACs prescription appropriateness and clinically relevant bleedings in hospitalized oldest-old patients with AF was also assessed. The cystatin-C-based formulas showed excellent concordance, and were the ones determining 1/3 of reallocation of patients to a worse class of eGFR as compared to CKD-EPI_Cr_. Bleeding events increased by almost 2–3% in patients with discordant eGFR between formulas, reallocated to a worse CKD stage. CKD-EPI_Comb_ resulted the best predictor of bleeding events with a non-linear relationship between eGFR value and bleeding events, which significantly increase with eGFR values between 25 and 36 ml/min and a trend towards statistical significance for values between 37 and 50 ml/min. These results are in line with the range of eGFR implicated in the modification of DOAC dosage, highlighting the importance of an appropriate eGFR evaluation in DOACs prescription. Accordingly, patients with inappropriate DOACs dosage/prescription showed a higher incidence of bleeding events compared to the counterparts.

In detail, considering CKD-EPI_Cr_ as the reference formula, almost one third of the patients with preserved eGFR (PMD group) moved to the MD group by utilizing BIS_1_ equation. The addition of cystatin-C in either CKD-EPI_Cr_ or BIS_1_ algorithms led to a further reallocation of patients to a worse eGFR group across all the classes of renal function, although with different extent. The changes observed in our study are in line with those described when switching from creatinine- to cystatin-C-based eGFR estimating formulas, in either home-dwelling or hospitalized frail, older subjects [13, 14, 29, 30].

The crucial importance of an accurate estimation of eGFR in older AF patients eligible to DOAC therapy derives from the elevated haemorrhagic risk encompassed by renal impairment, especially for those with severe dysfunction [6]. Interestingly, while introducing cystatin-C in the estimating formulas, the overall bleeding risk increased, although slightly, in patients reallocated to a worse CKD group and receiving inappropriate DOACs dosage/prescription. Given the lack of statistical significance, we could not perform the subsequent regression model. This finding agrees with the results of our extended paper [19], in which eGFR emerged as a risk factor but not an independent predictor of bleeding events. Accordingly, the overall bleeding risk did not significantly differ across the CKD stages, regardless the formula used, confirming what was already shown in our main study [19], where, although the risk of bleedings increased in patients with severely depressed renal function, only the history of previous clinically significant bleedings emerged as independent predictor of subsequent bleeding events [19]. Of note, CKD-EPI_Comb_ showed the highest accuracy in detecting bleeding events compared to other formulas. Moreover, the non-linear relationship between CKD-EPI_Comb_ and bleeding events, is in line with the ranges of eGFR suggested for DOACs dose adjustment (30–50 ml/min/1.73 m^2^).

However, a trend of eGFR worsening and bleeding risk increment, similar to that obtained with BIS_2_, was observed also using BIS_1_, the most reliable creatinine-based eGFR estimating formula in older patients [17, 31]. This finding is of particular importance, confirming that BIS_1_ could be considered as a reliable alternative to cystatin-C-based formulas.

To the best of our knowledge, this is the first study evaluating the degree of eGFR changes and the possible CKD stage reallocation, according to diverse eGFR estimating equations, along with the potential impact on clinically relevant bleeding, in a cohort of oldest-old, hospitalized AF patients receiving DOACs. Malavasi et al. carried out a similar study, but in a smaller cohort of younger in- and out-patients, mostly (76.9%) with preserved renal function (> 50 mL/min/1.73m^2^). Moreover, formulas containing serum cystatin-C value were never utilized, and the potential effect of CKD stage reallocation on bleeding risk not evaluated [32]. At odds with the latter study, we did not utilize the Cockcroft–Gault formula for estimating GFR, as recommended by current guidelines [10]. However, our patients were hospitalized for a wide range of acute illnesses, including frequently (43%) heart failure, which could lead to important weight variation due to fluid overload. Moreover, many of our patients were not easy to weight, due to disability or being bed-bound.

Overall, these data underline that in oldest old patients with AF the risk of DOAC overdosing due to a potentially inaccurate eGFR estimation should be carefully considered, and the choice of the best formula prioritised. KDIGO recommends the use of CKD-EPI_Cr_ equation [33], while randomized trials used the Cockcroft–Gault formula and many laboratories report eGFR calculated by MDRD. Our data suggest that estimating eGFR with cystatin-C could better select patients at risk of bleeding events, and, therefore, requiring closer clinical follow-up and caution in therapeutic choices. Accordingly, as shown in community dwelling elderly [13–15] Cystatin-C-based estimating formulas are the most reliable ones for identifying older patients with CKD at higher risk of complications [34, 35]. This finding may, at least in part, reflect the higher accuracy of Cystatin-C as filtration marker in comparison to creatinine, in older individuals. On the other hand, it should be considered that Cystatin-C levels may be influenced by different cardiovascular risk factors, such as obesity, diabetes mellitus, smoking habit, chronic inflammation and thyroid disease, whose prevalence increases in older people [4, 36, 37].

Even though the results of this study are promising, they should be seen in light of some limitations.

We acknowledge that a greater sample size of patients could have shown more definitive results. Indeed, it would be interesting to evaluate the risk of bleeding for each type of DOAC but, the cohort is too small to be split into smaller groups depending on the DOAC used. We acknowledge also that the lack of data on CG derived eGFR could represent a further limitation. Finally, the assessment of mortality across Tuscany was conducted through a regional administrative archive, which however does not provide the cause of death. Thus, we could not detect the amount of bleeding-related deaths.

## Conclusions

DOACs management requires careful estimation of renal function and the use of the Cockcroft–Gault equation has been traditionally suggested. However, the concordance between CG and other eGFR estimating formulas may change or be lost, mainly while using cystatin-C-based algorithms, leading to potential inappropriate DOACs dosage and therefore to increased risk of clinically relevant bleeding. Our data suggest cystatin-C-based eGFR estimating equations as the most accurate in stratifying older patients, but several confounding factors may limit their reliability and the use in clinical practice. Moreover, the creatinine-based BIS_1_ formula could be considered a good and reliable estimating equation comparable to CKD-EPI_Comb_, at least for patients with preserved renal function. However, strong evidences indicating the superiority of cystatin-C-based equations in short- and long-term prognosis of older AF patients receiving DOACs are lacking. Further, large randomized studies are needed to confirm cystatin-C-based eGFR estimating formulas as the most reliable for prescribing and dosing DOACs in older patients.

## Supplementary Information

Below is the link to the electronic supplementary material.

Supplementary file1 (DOCX 14 KB)

Supplementary file2 (DOCX 14 KB)

## Data Availability

The datasets generated during and/or analyzed during the current study are available from the corresponding author on reasonable request.
