# Supplementary material for: Different glomerular filtration rate estimating formula for prescribing DOACs in oldest patients: appropriate dosage and bleeding risk. Post hoc analysis of a prospective cohort
Source: Aging Clin Exp Res. 2021 Oct 18;34(3):591–8. doi: 10.1007/s40520-021-01986-w (PMC8894223; doi:10.1007/s40520-021-01986-w)
Supplement: Supplementary file 1 — Supplementary file1 (DOCX 14 KB) [file 40520_2021_1986_MOESM1_ESM.docx]

Table 2. Concordance of kidney function classes according to the diverse eGFR equations as assessed by Cohen’s Kappa coefficient.

|  | CKD-EPI_Cr_ | CKD-EPI_Comb_ | BIS_1_ | BIS_2_ | MDRD |
| --- | --- | --- | --- | --- | --- |
| CKD-EPI_Cr_ | X | 0.703 (0.669-0.736) | 0.795 (0.764-0.825) | 0.636 (0.600-0.673) | 0.915 (0.895-0.935) |
| CKD-EPI_Comb_ | X | X | 0.725 (0.692-0.758) | 0.868 (0.844-0.891) | 0.631 (0.595-0.667) |
| BIS_1_ | X | X | X | 0.741 (0.706-0.775) | 0.742 (0.707-0.777) |
| BIS_2_ | X | X | X | X | 0.575 (0.536-0.614) |
| MDRD | X | X | X | X | X |

eGFR estimated glomerular filtration rate; CKD-EPI_Cr_ Chronic Kidney Disease-Epidemiology Collaboration creatinine-based; CKD-EPI_Comb_ Chronic Kidney Disease-Epidemiology Collaboration creatinine-cystatin-C based; BIS_1_ Berlin Initiative Study creatinine; BIS_2_ Berlin Initiative Study creatinine-cystatin-C; MDRD Modification of Diet in Renal Disease
