# Supplementary material for: Different glomerular filtration rate estimating formula for prescribing DOACs in oldest patients: appropriate dosage and bleeding risk. Post hoc analysis of a prospective cohort
Source: Aging Clin Exp Res. 2021 Oct 18;34(3):591–8. doi: 10.1007/s40520-021-01986-w (PMC8894223; doi:10.1007/s40520-021-01986-w)
Supplement: Supplementary file 2 — Supplementary file2 (DOCX 14 KB) [file 40520_2021_1986_MOESM2_ESM.docx]

**Supplementary Table 1 A**. Number of patients taking inappropriate dosage of DOAC in comparison to CKD-EPI_cr_

|  | **MDRD** | **BIS1** | **CKD-EPI_Comb_** | **BIS2** |
| --- | --- | --- | --- | --- |
| **Dabigatran 150 mg BID** | 0/22 | 4/22  (18.2%) | 12/22  (54.5%) | 8/22  (36.4%) |
| **Apixaban 5 mg BID** | 0 /113 | 1/113  (0.9%) | 31/113  (27.4%) | 7/113  (6.2%) |
| **Rivaroxaban 20 mg** | 0/80 | 23/80  (28.7%) | 48/80  (60.0%) | 39/80  (48.7%) |
| **Edoxaban 60 mg** | 0/8 | 2/8  (25.0%) | 5/8  (62.5%) | 4/8  (50.0%) |

**Supplementary Table 1 B.** Number of patients who should discontinue DOAC therapy in comparison to CKD-EPI_cr_

|  | **MDRD** | **BIS1** | **CKD-EPI_Comb_** | **BIS2** |
| --- | --- | --- | --- | --- |
| **Dabigatran 110 mg BID** | 4/230  (1.7%) | 6/230  (2.6%) | 27/230  (11.7%) | 15/230  (6.5%) |
| **Apixaban 2.5 mg BID** | 1/165  (0.6%) | 0/165 | 7/165  (4.3%) | 0/165 |
| **Rivaroxaban 15 mg** | 1/207  (0.5%) | 1/207  (0.5%) | 16/207  (7.7%) | 0/207 |
| **Edoxaban 30 mg** | 0/16 | 0/16 | 1/16  (6.2%) | 0/16 |
